# Parental awareness, knowledge, and attitude about shaken baby syndrome in Riyadh, Saudi Arabia: a cross-sectional study

**DOI:** 10.11604/pamj.2022.41.327.33708

**Published:** 2022-04-22

**Authors:** Hanan Ibrahim AlOmran, Zaid Ibrahim AlKharaan, Khalid Mubarak AlDawsari, Osamah Zeid AlDakkan, Hamad Mesfer AlAtif, Manal Zaher Elewa Mohamed

**Affiliations:** 1 Department of Pediatric, College of Medicine, Prince Sattam Bin Abdulaziz University, Al-Kharj, Saudi Arabia

**Keywords:** Awareness, attitude, knowledge, parents, shaken baby syndrome

## Abstract

**Introduction:**

shaken baby syndrome (SBS) is an abusive head trauma inflicted on infants and young children. Injuries induced by shaking can result in death or permanent neurologic disability. It is difficult to know the exact number of SBS cases per year because many cases of SBS are not reported and/or never receive a diagnosis. From a public health perspective, creating greater awareness about SBS is important. Previous studies have revealed poor awareness and knowledge about shaken baby syndrome. In this study our aims to investigate the awareness, knowledge level, and attitude regarding shaken baby syndrome and to highlight the sources and factors associated with SBS knowledge among parents of the pediatric population in Riyadh, Saudi Arabia.

**Methods:**

a cross-sectional study was conducted between January 2021 and September 2021. A validated electronic questionnaire was distributed among parents of the pediatric population in Riyadh, Saudi Arabia using the convenient random sampling method; SPSS version 22 was used to analyze the collected data.

**Results:**

the study involved 577 participants; 59.8% were mothers and 96.5% were Saudis. A total of 32.1% had previously heard about SBS. The overall knowledge mean score was low (2.95 + 1.74), and attitude was positive among 82.5% of the participants. The factors significantly associated with knowledge level were gender, marital status, and occupation.

**Conclusion:**

the participants had poor knowledge and awareness about SBS, but, despite that, they expressed a positive attitude toward learning more about it. This should prompt health authorities to increase efforts to improve public awareness and knowledge about SBS.

## Introduction

Shaken baby syndrome (SBS) is a term often used by physicians and the public to describe abusive head trauma inflicted on infants and young children [1]. In 1984, Ludwig and Warman first published the term “shaken baby syndrome” in their review of 20 infants and young toddlers injured by shaking, none of whom showed evidence of impact injury to the head [2]. Injuries induced by shaking can result in death or permanent neurologic disability, including static encephalopathy, mental retardation, cerebral palsy, cortical blindness, seizure disorders, and learning disabilities [1].

It is difficult to know the exact number of SBS cases per year because many cases of SBS are not reported and/or never receive a diagnosis. However, a study of SBS cases in North Carolina suggests that as many as three to four children a day experience severe or fatal head injury from child abuse in the United States [3]. The most common trigger for shaking a baby is inconsolable or excessive crying in association with outside stressors created by work, social, and/or financial challenges. Babies less than one year of age (with the highest risk period at two to four months) are at greatest risk for SBS because they cry longer and more frequently and are easier to shake than older and larger children [4]. Shaken baby syndrome injuries have been reported in children up to the age of five [5].

From a public health perspective, creating greater awareness about SBS is important. This awareness includes understanding the dangers of violently shaking a baby, the risk factors associated with SBS, the triggers for it, and ways to prevent it. Greater awareness may help reduce the number of babies affected by SBS [6]. A 2013 study that assessed Turkish parents´ knowledge of SBS reported that 50.3% had no idea that shaking a baby is dangerous and 24% thought that shaking would not be dangerous [7]. In 2015, an Irish study reported that half of the participants had no prior knowledge of shaken baby syndrome and that educating the parents may help to avoid inappropriate treatment of crying infants, including shaking babies [8]. A 2018 Tabuk study reported that 67.39% of participants parents had no prior knowledge about the risks of SBS [9]. Finally, in a 2019 German study, 40% of participants had no prior knowledge about abusive head trauma (AHT) and shaken baby syndrome [10].

With an aim toward prevention, the American Academy of Pediatrics recommends increased awareness and education programs for parents and caregivers about the danger of shaking babies and safe approaches to calm and cope with a crying infant. Low awareness and knowledge about shaken baby syndrome can result in permanent neurological damage, which could lead to tremendous family and social costs. In contrast, having a high level of awareness about shaken baby syndrome should help to prevent the development of its consequences. In this study, our aim is to investigate the level of awareness and knowledge about shaken baby syndrome and to highlight the sources and factors associated with SBS knowledge among parents of the pediatric population in Riyadh, Saudi Arabia.

## Methods

**Study design:** a cross-sectional, descriptive, questionnaire-based study that was conducted between January and September 2021 by distributing validated electronic questionnaires of multiple-choice questions to investigate the awareness, knowledge level, and attitude regarding shaken baby syndrome and to highlight the sources and factors associated with SBS knowledge among parents of the pediatric population in Riyadh, Saudi Arabia.

**Study setting and study subjects:** Riyadh is the capital of Saudi Arabia and one of the largest cities on the Arabian Peninsula. it had a population of 7.6 million people in 2019, making it the most populous city in Saudi Arabia, third most populous in the Middle East, and 38^th^ most populous in Asia. The targeted study subjects and inclusion criteria were all the parents of the pediatric age group living in Riyadh, Saudi Arabia, and willing to participate in the study. The exclusion criteria were people without children and parents living outside Riyadh.

**Data collection, and questionnaire validation:** a validated electronic questionnaire was distributed on social media platforms to the target population using the convenient random sampling method and parents were kindly asked to complete it. A questionnaire was developed and validated in multiple steps. First, to ensure that the content of the survey was serving the study aims appropriately and covering the topic well, the questionnaire was constructed by a pediatric consultant who is also a research expert. Then, three faculty physicians who have subject matter and research experience confirmed the content validity. Third, pre-testing was conducted on 20 individuals who were excluded from the main study. Brief cognitive interviewing was then undertaken with these individuals to explore any problem areas in the formulation and order of questions, appropriateness of length, and relevant questionnaire processes. The questionnaire was first written in English then translated by a linguist into Arabic (the local language for target population), then translated back into English to ensure accurate usage of words and grammar. It covered four sections: socio-demographic characteristics of participants (7 items), parents´ knowledge about SBS (4 items), parents´ practice toward SBS (1 item), and parents´ attitude toward SBS (3 items).

**Sample size:** sample size calculation was performed using the formula:


$$n=\frac{z^{2}pq}{d^{2}}$$

The confidence level was set at 95%, the estimated proportion was set at 50%, and the level of precision was 5%; sample size was calculated to be 385. However, more participants were enrolled to secure more accurate results.

**Data analysis:** after data were entered and extracted using Microsoft Office Excel, they were revised, coded, and fed to statistical software IBM SPSS version 22 (SPSS, Inc, Chicago, IL). All statistical analysis was done using two-tailed tests. A P-value of less than 0.05 was considered statistically significant. For knowledge items, each correct answer was scored one point and the total summation of the discrete scores of the different items was calculated. Knowledge score was calculated as follows: Each correctly answered item was given one point, the maximum possible score was eight, and the minimum possible score was 0, by using 60% cut-off points, with <60% considered a poor knowledge level (0-4 points) and >60% considered a good knowledge level (5-8 points). Descriptive analysis based on frequency and percent distribution was done for all variables including sociodemographic data. In addition, descriptive frequencies were tabulated for knowledge items, participants´ practice, and attitude. Cross-tabulation was used to assess distribution of knowledge and attitude levels according to participants´ sociodemographic data. An independent t-test and ANOVA test were used to test for association. A Chi-square test was also used to test for association between categorical variables.

**Ethical consideration:** this study was ethically approved by Prince Sattam Bin Abdelaziz University´s Scientific Research Ethics Committee. REF: PSAU/COM/RC/IRB/P/88. And the questionnaire included a letter explaining the study´s nature and purpose. Participation consent was taken prior to beginning the questionnaire. Confidentiality was maintained as the questionnaires did not include any names or numbers and the data were only accessible to the authors.

## Results

The study included 577 respondents, of whom 345 (59.8%) were mothers. The majority were 40 years old and older. Exactly 557 (96.5%) were Saudis, and 562 (97.4%) were married. Three hundred forty (58.9%) participants had a bachelor´s degree, and 412 (71.4%) were employees. Additional details on sociodemographic data are presented in Table 1. In this study, we found 185 (32.1%) of participants were aware about shaken baby syndrome, while 392 (67.9%) were not. Participants´ sources of information about shaken baby syndrome were primarily from the internet and social media (55.7%), followed by family members and friends (26.5%) and health workers (13.5%). Only 33.6% of participants recognized shaking a baby as a harmful behavior, and 28.8% knew that shaking a baby can lead to death; 89.4% knew that shaken baby syndrome can be prevented (Table 2). In total, 18.7% had a good knowledge level about shaken baby syndrome (mean of 2.95 + 1.74).

**Table 1:** socio-demographic profile of the participants (n = 577)

Demographical characteristics	n	%
**Gender**		
Mother	345	59.80
Father	232	40.20
**Age**		
Less than 20 years old	19	3.30
Between 20 to 29 years old	139	24.10
Between 30 to 39 years old	189	32.80
40 years or older	230	39.90
**Nationality**		
Saudi	557	96.50
Non-Saudi	20	3.50
**Marital status**		
Married	562	97.40
Divorced	12	2.10
Widowed	3	0.50
**Education**		
High school education or lower	99	17.20
Diploma	73	12.70
Bachelor	340	58.90
Postgraduate degree	65	11.30
**Occupation**		
Employee	412	71.40
Housewife	140	24.30
Student	25	4.30
**How many children you have?**		
1 - 4 children	406	70.40
5 - 7 children	145	25.10
More than 7 children	26	4.50

**Table 2:** participants' knowledge about shaken baby syndrome (n = 577)

Question	n	%
**Q1/ Do you think a shake the baby is**		
Non-harmful	56	9.7
Maybe harmful	242	41.9
Harmful	194	33.6
I don't know	85	14.7
**Q2/ What do you think the sequence of shaken baby syndrome? (More than one answer can be chosen)**		
Blindness	65	11.3
Cerebral hemorrhage	237	41.1
Learning difficulties	157	27.2
Behavior change	227	39.3
Coma	139	24.1
No sequencies	145	25.1
**Q3/ Do you think a shake infant leads to death?**		
Yes	166	28.8
No	411	71.2
**Q4/ Do you think we can prevent the shaken baby syndrome?**		
Yes	516	89.4
No	61	10.6
**Knowledge score (0-8)**		
Mean	2.95	
Standard deviation	1.74	
**Knowledge level**		
Poor (0-4)	469	81.30
Good (5-8)	108	18.70

**Figure 1:**
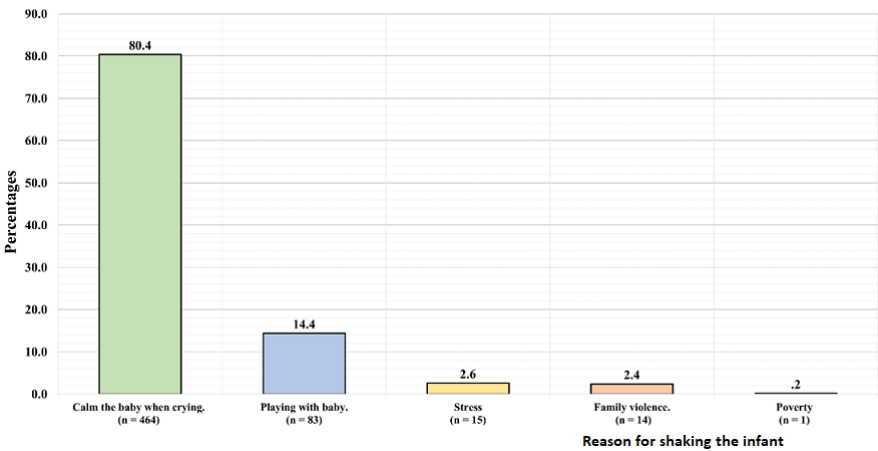
in your opinion why someone shakes an infant?

Regarding participants´ attitude toward shaken baby syndrome (Table 3), 476 (82.5%) had a positive attitude (they wanted to know more about shaken baby syndrome). Of those who wanted to know more about shaken baby syndrome, 201 (42.2%) stated they wanted to learn through the internet and social media, 128 (26.9%) through doctors or medical staff, 122 (25.6%) through an awareness campaign, and 25 (5.3%) from medical books; the majority (38.7%) wanted to receive the information during pregnancy. As for the parents´ practice related to shaken baby syndrome when their baby cries, 328 (56.8%) of the participants would carry their baby, 132 (22.9%) would calm the baby by shaking it, 91 (15.8%) would pat the baby´s back to calm it, 19 (3.3%) would ask family and friends for support, and 7 (1.2%) would not do anything (Table 3). When the participants were asked whether crying is a major cause for shaking a baby, 329 (57%) answered yes, while 248 (43%) answered no. Regarding the reasons for shaking a baby, the majority of our participants (464; 80.4%) answered it was to calm the baby when it was crying (Figure 1).

**Table 3:** participants' attitude and practice toward shaken baby syndrome (n = 577)

Question	n	%
**Q1/ Do you want to know more about shaken baby syndrome?**		
Yes, I want	476	82.5
No, I don't want to	101	17.5
**Q2/ If yes through which sources? (n = 476)**		
Doctor or medical staff during the vaccination period	128	26.9
Internet and social media	201	42.2
Awareness campaign	122	25.6
Medical books and bulletins	25	5.3
**Q3/ What is the preferred period of time you want to receive the information about shaken baby syndrome? (n = 476)**		
Before pregnancy period	113	23.7
During pregnancy period	184	38.7
One week after delivery	82	17.2
During vaccination visits for baby	97	20.4
**Parents practice**		
**What you do when your baby crying? (n = 577)**		
I'm calming him by shaking	132	22.9
I'm carrying him	328	56.8
I'm patting on his/her back	91	15.8
I'm asking for help from family member or friends	19	3.3
I'm not doing anything	7	1.2

Table 4 demonstrates the distribution of parents´ knowledge level by their personal data. Parents´ gender was significantly associated with their knowledge level (p = 0.04), and mothers had a higher rate of good knowledge compared to fathers (21.4% vs 14.7%). Marital status was also significantly related to knowledge level (p = 0.042), and the widowed had the highest rate of good knowledge (66.7%), followed by divorced (33.3%), and, finally, married people (18.1%). Occupation was also found to be significantly correlated with knowledge level (p = 0.02), and students had a notably higher rate of good knowledge compared to employees and housewives (40% vs. 17.5% and 18.6%, respectively). Age, nationality, education, number of children, and attitude (wanting to learn more about shaken baby syndrome) were not significantly associated with knowledge about shaken baby syndrome. Table 5 demonstrates factors associated with a positive attitude regarding learning about SBS. Only education was found to be significantly associated with positive attitude (p < 0.001), and participants with postgraduate degrees had the highest rate of positive attitude (95.4%), followed by those with bachelor´s degrees (84.7%).

**Table 4:** factors associated with knowledge toward shaken baby syndrome

Factor	Knowledge level		P-value
	Poor	Good	
**Gender**			0.04*
Mother	271 (78.6%)	74 (21.4%)	
Father	198 (85.3%)	34 (14.7%)	
**Age**			0.416
Less than 20 years old	17 (89.5%)	2 (10.5%)	
Between 20 to 29 years old	116 (83.5%)	23 (16.5%)	
Between 30 to 39 years old	156 (82.5%)	33 (17.5%)	
40 years old or more	180 (78.3%)	50 (21.7%)	
**Nationality**			0.309
Saudi	451 (81%)	106 (19%)	
Non-Saudi	18 (90%)	2 (10%)	
**Marital status**			0.042*
Married	460 (81.9%)	102 (18.1%)	
Divorced	8 (66.7%)	4 (33.3%)	
Widowed	1 (33.3%)	2 (66.7%)	
**Education**			0.761
Secondary and below	82 (82.8%)	17 (17.2%)	
Diploma	62 (84.9%)	11 (15.1%)	
Bachelor	272 (80%)	68 (20%)	
Postgraduate	53 (81.5%)	12 (18.5%)	
**Occupation**			0.02*
Employee	340 (82.5%)	72 (17.5%)	
Housewife	114 (81.4%)	26 (18.6%)	
Student	15 (60%)	10 (40%)	
**How many children you have?**			0.064
1 - 4 children	340 (83.7%)	66 (16.3%)	
5 - 7 children	109 (75.2%)	36 (24.8%)	
More than 7 children	20 (76.9%)	6 (23.1%)	
**Do you want to know more about shaken baby syndrome?**			0.979
Yes, I want	387 (81.3%)	89 (18.7%)	
No, I don't want to	82 (81.2%)	19 (18.8%)	

* Significant at level 0.05

**Table 5:** distribution of parents' attitude level by their personal data

Factor	Parents' attitude toward SBS (wanting to learn more vs not)		P-value
	Negative	Positive	
**Gender**			0.593
Mother	287 (83.2%)	58 (16.8%)	
Father	189 (81.5%)	43 (18.5%)	
**Age**			0.117
Less than 20 years old	12 (63.2%)	7 (36.8%)	
Between 20 to 29 years old	113 (81.3%)	26 (18.7%)	
Between 30 to 39 years old	156 (82.5%)	33 (17.5%)	
40 years or older	195 (84.8%)	35 (15.2%)	
**Nationality**			0.765
Saudi	460 (82.6%)	97 (17.4%)	
Non-Saudi	16 (80%)	4 (20%)	
**Marital status**			0.195
Married	461 (82%)	101 (18%)	
Divorced	12 (100%)	0 (0%)	
Widowed	3 (100%)	0 (0%)	
**Education**			< 0.001*
High school education or lower	75 (75.8%)	24 (24.2%)	
Diploma	51 (69.9%)	22 (30.1%)	
Bachelor	288 (84.7%)	52 (15.3%)	
Postgraduate degree	62 (95.4%)	3 (4.6%)	
**Occupation**			0.252
Employee	346 (84%)	66 (16%)	
Housewife	109 (77.9%)	31 (22.1%)	
Student	21 (84%)	4 (16%)	
**How many children you have?**			0.470
1 - 4 children	340 (83.7%)	66 (16.3%)	
5 - 7 children	115 (79.3%)	30 (20.7%)	
More than 7 children	21 (80.8%)	5 (19.2%)	

* Significant at level 0.05

## Discussion

Shaken baby syndrome is a serious health condition that can result in permanent brain damage or death. Since this can be preventable by creating greater awareness and knowledge about SBS, it is important to investigate whether parents have a satisfactory level of awareness and knowledge about SBS. However, little research has been published regarding awareness, knowledge, and attitude about shaken baby syndrome.

In our study, we found that awareness about SBS was inadequate and only 185 (32.1%) participants had previously been aware of SBS. This finding is consistent with the results found in a similar study conducted in a northern region of Saudi Arabia, which also reported a low level of awareness [9]. This low rate of awareness could be attributed to the fact that there are not enough educational programs and awareness campaigns targeting parents about such topics. In contrast, a study conducted in Germany [10] reported that 59.4% of the participants had previously heard about SBS. This difference might be explained by the higher number of awareness campaigns about SBS in western countries.

We also found in this study that the participants´ overall knowledge about SBS was low. The previous studies did not assess the overall knowledge level in the same manner as this study, but the results of this study reflect a large defect in participants´ knowledge about SBS. Moreover, in the study conducted by Dias *et al*. [11], knowledge about shaken baby syndrome was exceptionally high, which was, according to the authors, attributed to the study being conducted after 1) a famous incident of shaken baby syndrome being well covered in the media and 2) the recent launching of two prevention programs in the region. This highlights the importance of media and educational programs in raising awareness about medical topics.

The main source of SBS information, as found in our study, was the internet and social media followed by family and friends and then healthcare workers, which is consistent with the results of previous studies [10]. This is as can be expected as the internet and social media are the leading source of knowledge in many aspects of life. Only 166 (28.8%) participants recognized death as a possible result of shaken baby syndrome, which is not surprising, considering that only 32.1% of the participants had heard about shaken baby syndrome. In Berthold *et al*. [10] work, 90.8% recognized death as a possible consequence of shaken baby syndrome. This difference might be reflecting the wide knowledge gap between western and eastern countries regarding shaken baby syndrome, which necessitates an educational program to raise awareness about this topic in the studied region. However, this difference could have been overestimated, as Berthold *et al*. [10] asked this question only to participants who reported hearing about shaken baby syndrome before.

Although the knowledge level was low in this study, a high positive attitude was observed among the participants, as 82.5% expressed their desire to learn more about SBS. The most preferred way of learning was through the internet and social media, and the most preferred time was during the antenatal period. This is inconsistent with Berthold *et al*. [10], where the preferred method of learning was written material and not online webpages. Mann *et al*. [8] reported a similar preferred timing of learning, which was during the antenatal period, and the preferred method of learning was reading material.

As for the factors associated with knowledge level about shaken baby syndrome, this study found gender, marital status, and occupation to be significantly associated with knowledge level. The gender association with knowledge was also previously reported by Berthold *et al*. [10], Dias *et al*. [11], Bechtel *et al*. [12], and Simonnet *et al*. [13], where females had significantly higher knowledge about shaken baby syndrome compared to males. Bechtel *et al*. [12] also found a significant relationship between knowledge about SBS and education level, which was not present in this study. An association between low socio-economic status and low educational level with all types of maltreatment toward children has been previously identified in multiple studies [14-17]. The relationship between education level and knowledge about SBS was not observed in this study; this could be due to the fact that the overall knowledge was low, which might have blurred the differences between the different educational levels.

## Conclusion

In conclusion, our findings show that the parents´ knowledge about SBS was inadequate. However, a high positive attitude was observed among the participants. Positive attitude alone, however, does not actually affect the parents´ practice. Therefore, the responsible health authorities should increase efforts to improve public awareness and knowledge about SBS to prevent its consequences.

**Recommendations:** we first recommend conducting research on the prevalence of shaken baby syndrome in Saudi Arabia to measure the scale of the problem in the country. Furthermore, there should be an implementation of educational programs about shaken baby syndrome and the measures to prevent it that target pregnant women during the antenatal period, since the overall level of knowledge was poor.

**Limitations:** study participants were chosen from one geographical area in Saudi Arabia, which means that the current results cannot be generalized across the country; a sample size including participants from all the country´s areas would provide a better view on the topic. However, this study included a large number of participants in order to counteract this potential bias, and the results were similar to previous findings in the literature.

### What is known about this topic

- Shaken baby syndrome (SBS) is an abusive head trauma inflicted on infants and young children;
- Injuries induced by shaking can result in death or permanent neurologic disability, including static encephalopathy, mental retardation, cerebral palsy, cortical blindness, seizure disorders, and learning disabilities;
- The most common trigger for shaking a baby is inconsolable or excessive crying, with an aim toward prevention, the American Academy of Pediatrics recommends increased awareness and education programs for parents and caregivers about the danger of shaking babies and safe approaches to calm and cope with a crying infant.

### What this study adds

- The study showed there was poor awareness about SBS in Riyadh city, Saudi Arabia, but despite that there was a positive attitude toward learning more about it, these findings should prompt health authorities to increase efforts to improve public awareness and knowledge about SBS.
